# Diseases Admitted under the Care of the Physicians of the Westminster Hospital, from the 18th of April to the 21st of May, 1800

**Published:** 1800-06

**Authors:** 


					State of Difeafes in London. 50$
]}ifeafes admitted under the Care of the Phyjicians of the Wcjlminjler
Hofpital, from the 18th of April to the Z\Ji of May, 1800.
Continued Fever - - - 10
Jntermittents - - - - j
Pleurify ------ 4
Meafles ------ 1
Amenorrhoea - , - - - 7
Anafarca - - - - 4
Aphthae - 1 - - - - 1
Afcites ------ I
Afthenia - - - - - 9
Cephalaea ----- 5
Colic - - - - - 3
Convulfions - - - - - 2
Cough - - - - - - rl
Diarrhoea - - - - r 5
| Dyfpepfia
506 State of Difeafes in London.
Dyfpepfia - - - - 2
Dyluria ------ 3
Enterodynia - - - - 6
Erythema - - - - - - 2
Gaftrodynia ----- 3
Hasmoptyfis ----- 3
Haemorrhois - *? - 2
Hypochondriafis -- - - i
Impetigd - - - ' - - 2
Itch - ------ 4
Lepra Graec - - - - 1
Lichen ------ i
Menorrhagia - - - - 2
Obftipatio ----- j
Paralyfis ------ 2
Palpitation ----- 1
Phthifis ------ 2
Rheumatifm - - - 9
Sciatica ------ 3
Struma - - - - - - ? 3
Worms ------ 2

				

